# A paired analysis of mercury among non-invasive tissues in Mexican free-tailed bats (*Tadarida brasiliensis*) to inform conservation monitoring

**DOI:** 10.1007/s10646-025-02928-2

**Published:** 2025-07-02

**Authors:** Molly C. Simonis, Kimberlee Whitmore, Kristin E. Dyer, Meagan Allira, Bret Demory, Matthew M. Chumchal, Daniel J. Becker

**Affiliations:** 1 School of Biological Sciences, University of Oklahoma, Norman, OK USA; 2 College of Forestry, Wildlife, and Environment, and College of Veterinary Medicine Department of Pathobiology, Auburn University, Auburn, AL USA; 3 Department of Biology, Texas Christian University, Fort Worth, TX USA

**Keywords:** Bioaccumulation, Feces, Fur, Risk assessment, Total mercury

## Abstract

Contaminants can harm wildlife. However, measuring wildlife exposure to contaminants can be challenging due to accessibility of species and/or sampling tissue matrices needed to assess wildlife health risks. For example, in bats and other taxa that use roosts, collecting feces under colonies minimizes disturbance to species of conservation concern, but fecal contaminant concentrations may not directly correlate with tissue contaminant concentrations. Thus, there is a need for quantifying contaminant exposure relationships between sample matrices for initial risk assessments to address wildlife health and conservation needs. Our goal was to assess the relationship between fecal and fur total mercury (THg) concentrations. We collected paired feces and fur samples (*n* = 48) from Mexican free-tailed bats (*Tadarida brasiliensis*) in summer 2023 in western Oklahoma at a maternity roost. At the individual level, we found no correlation between fecal and fur THg. However, at the population level, fur THg concentrations were on average 6.06-times greater than fecal THg concentrations. We conclude that although fecal THg cannot serve as a proxy of individual bat fur THg, population-level differences could be used cautiously to estimate mean fur THg concentrations from under-roost feces and motivate individual-level sampling to assess health impacts. We encourage continued research across other insectivorous bat species and sites for determining THg relationships across tissues and initial risk assessments with minimal disturbance to species of conservation concern when a contaminant point source is not yet known.

## Introduction

Exposure to high concentrations of potentially toxic elements such as mercury (Hg) can harm wildlife (Wolfe et al. 1998; Scheuhammer et al. 2007; Tan et al. 2009; Chételat et al. 2020). For example, at elevated concentrations, Hg can reduce reproductive success, cause immunotoxicity, and damage genetic material (Wolfe et al. 1998; Calao-Ramos et al. 2021; Teitelbaum et al. 2022; Leaphart et al. 2025). To determine how contaminant exposure affects wildlife health, researchers must sample the appropriate tissue matrix (e.g., fur, blood, feces) that best relates to the health outcome of interest, as different matrices indicate different contaminant exposure and/or bioaccumulation timelines (Chételat et al. 2020). For example, Hg concentrations in fur represent longer-term exposure (e.g., weeks to months depending on molting cycles), while Hg concentrations in feces represent more acute exposure/ingestion of Hg (e.g., days to weeks depending on metabolism and excretion efficiency; Chételat et al. 2020). Some matrices can be sampled using minimally invasive procedures that do not require handling wildlife (e.g., collection of feces under bat roosts) while sampling other matrices require handling and disturbing (i.e., collecting fur), and therefore may not possible for species of conservation concern. There remains a need to assess risks of long-term contaminant exposure with minimal disturbance to sensitive wildlife species.

Bats are a highly diverse group of mammals that are of special interest to conservation (Kunz and Fenton 2003). Bats are important bioindicator species due to their long lifespans (e.g., the oldest known wild bat was 41 years old; Podlutsky et al. 2005), slow birth rates (one to two pups per year; Wilkinson and South 2002), high mobility through flight, diverse habitat use, and—for insectivorous bats—feeding at high trophic levels (Jones et al. 2009; Zukal et al. 2015; Russo et al. 2021). In North America, many bats are threatened by white-nose syndrome (Blehert et al. 2009; Lorch et al. 2011), which has driven population declines of multiple species (Frick et al. 2015; Cheng et al. 2021). Exposure to contaminants such as Hg can increase individual bat infection risk (Becker et al. 2021) and also shape infection prevalence through diverse feedback loops (Cable et al. 2021; Becker et al. 2024).

Depending on the bat species, collecting samples needed to quantify health can be complicated (Giles et al. 2021), particularly when aiming to measure contaminant concentrations. For example, sampling pooled bat feces or urine under roosts or outside cave entrances may be most appropriate for species of conservation concern to minimize disturbance (Walker et al. 2016; Giles et al. 2021); however, feces can only provide insight into short-term contaminant exposure, and pooled sampling is not traceable to individuals. Estimating long-term contaminant exposure is better measured by sampling fur, which is strongly correlated with Hg concentrations in blood and indicative of age-related bioaccumulation (Wada et al. 2010; Yates et al. 2014). In cases where sampling fur from bats is disruptive to population health (e.g. disturbance of threatened and endangered species), researchers could collect under-roost fecal samples as a preliminary screening assessment, and only use more intrusive individual-level collection techniques, like fur sampling, if contaminant concentrations in feces were high enough to justify additional sampling. However, formal methods for converting fecal contaminant concentrations to contaminant concentrations in fur are lacking, hindering our ability to rapidly identify bat health risks from under-roost samples and, in turn, delaying conservation actions.

Mexican free-tailed bats (*Tadarida brasiliensis*), a common insectivorous bat across North America, migrate long-distances annually in spring and fall between northern and southern portions of their range (Wilkins 1989). These bats are highly valuable to conservation due to the ecosystem services they provide throughout their range. *T. brasiliensis* provide important nutrient transfer to soils across the USA and Mexico from their guano, particularly in the USA where they form large maternity colonies in spring and summer (thousands to millions of individuals; Bernardo and Cockrum 1962). In the USA, *T. brasiliensis* eat up to 79% of their body weight in agricultural pests (about 8.1 g of insects per individual) each night during peak lactation (Kunz et al. 1995), saving up to $1.7 million in agricultural pest control each year (Cleveland et al. 2006). Finally, *T. brasiliensis* are valuable to the ecotourism industry, with public viewing of nightly maternity colony emergence throughout the southwestern USA estimated to bring in over $6.5 million to local economies (Bagstad and Wiederholt 2013), highlighting their high intrinsic and monetary value to the public.

In this study, we assessed the individual- and population-level relationships between total Hg (THg) concentrations in feces and fur of *T. brasiliensis*. We predicted that THg concentrations in fur and feces would be positively associated but would be greater in fur, owing to differences in bioaccumulation timelines associated with repetitive tissue turnover (fur) versus excretion (feces). Our aim was to inform whether fecal Hg could serve as an initial substitute for estimating fur Hg, such as in cases where under-roost sampling is the primary option for quantifying contaminants in bat populations.

## Materials and methods

### Study site and bat capture

As part of a larger study of bat migration, immunity, and infection (Becker et al. 2025), we quantified Hg concentrations in *T. brasiliensis* at the Selman Bat Cave in Woodward County, Freedom, Oklahoma, USA. We captured 48 bats on 16 June, 17 June, and 18 July 2023. Bats were captured with hand nets upon emergence and temporarily held in individual cloth bags. Because most *T. brasiliensis* arrive at Selman Bat Cave in April following migration from Mexico (Glass 1982), capturing bats in June and July ensured (1) bats were foraging within the local landscape for multiple months and (2) fur had grown during their summer residency (Constantine 1957; Fraser et al. 2013). From each bat, we collected paired samples of 1–2 fecal pellets (opportunistically collected from bat holding bags) and fur trimmed from the mid-dorsal region. We weighed each individual to the nearest gram and identified bats by sex, reproductive status (i.e., non-reproductive, testes descended, pregnant, lactating, and post-lactating), and age (i.e., juvenile, subadult, and adult; Morgan et al. 2019). All bats were released after sampling. Feces per individual were stored in 1 mL 95% ethanol in a portable −20 °C cooler, and individual fur samples were stored in 1.5 mL microcentrifuge tubes at room temperature. Samples were transported to the University of Oklahoma and stored at the same temperatures until processing.

### THg analyses

At the University of Oklahoma, we prepared individual fur samples to perform THg analyses (MeHg and inorganic Hg combined). The mass of fur used ranged from 0.8–4.9 mg, with over 75% of samples having at least 4 mg fur (11 samples had smaller volumes). Once weighed, fur samples were heat treated at 60 °C for 30 min.

At Texas Christian University, feces and fur samples were further dried in a 60 °C drying oven for at least 48 h. All fecal and fur samples were analyzed for THg using direct Hg analysis (Nippon MA-3000, NIC), which uses thermal decomposition, gold amalgamation, and atomic absorption spectroscopy (US Environmental Protection Agency 1998). The analyzer was calibrated using a liquid standard (AGS Scientific) and quality assurance included solid reference samples (National Research Council of Canada Institute for National Measurement Standards) and duplicate samples. Reference samples (TORT-3 and PACS-2) were analyzed every 20 samples, and the mean recovery percentage for TORT-3 was 99.1 ± 2.42% (*n* = 13). The mean recovery percentage for PACS-2 was 98.9 ± 5.74% (*n* = 13). Duplicate samples of fur were analyzed every 20 samples, and the mean relative percent difference was 1.86 ± 1.78% (*n* = 6). Duplicate samples of feces were analyzed every 20 samples, and the mean relative percent difference was 7.59 ± 3.24% (*n* = 2). The limit of detection was determined by utilizing the measured limit of blank and test replicates of a sample known to contain a low concentration of analyte (Armbruster and Pry 2008). All samples were above the limit of detection of 0.02 ng THg.

### Statistical analyses

For statistical analyses (and forthcoming results), we included all adults, juveniles, and unknown demographic bats. The sampling period represents the reproductive season where body mass is highly variable due to varying energy requirements throughout female reproduction and juvenile growth, such that body mass would not directly reflect food intake (Kunz et al. 1995; Kunz and Robson 1995) and, thus, Hg bioaccumulation. Additionally, while insectivorous bats differ in THg concentrations between ages and reproductive statuses (Yates et al. 2014; Kieffer et al. 2023), sample sizes were not evenly distributed for direct comparisons. Similarly, analysis of all bats is comparable to what would be included in true under-roost sampling, where bat identity is not known.

We performed all statistical analyses and data visualization in the statistical environment R version 4.3.1 ‘Beagle Scouts’ (R Core Team 2023). All data visualizations were created using the package *ggplot2* (Wickham 2016). We created a generalized linear mixed effects model (GLMM) with a Gamma family and log link, using the function glmer() from the *lme4* package (Bates et al. 2015), to first test for a relationship between individual fecal and fur THg concentrations (ug/g dry). The GLMM was fit to fur THg concentrations as a function of fecal THg concentrations, with sample date as a random effect. We used Type II ANOVA tests with the function Anova() from the *car* package (Fox and Weisberg 2019) and obtained marginal and conditional R^2^ using the function r.squaredGLMM() from the *MuMIn* package (Bartoń 2023).

To next determine differences between paired bat fecal and fur Hg concentrations at the population level, we created another GLMM with a Gamma family and log link. This GLMM was fit to THg concentrations (μg/g dry) as a function of sample matrix (feces or fur). with random effects of individual bat and date of sample collection. We tested differences between paired samples using a Type II ANOVA, and we used the *emmeans* package and its self-titled function to obtain estimated marginal means of fur and fecal THg (Lenth 2021). We again obtained marginal and conditional *R*^*2*^.

## Results

We captured 48 *T. brasiliensis* representing 44 females (five non-reproductive, nine pregnant, 25 lactating, five post-lactating), three males (all non-reproductive juveniles), and one bat whose demographics were unknown. Of the captured bats that were female, two were juveniles, one was a subadult, and 41 were adults. We captured most bats for paired feces and fur samples during June (18 June 2023: *n* = 12; 19 June 2023: *n* = 13), with the remaining bats sampled on 18 July 2023 (*n* = 22). Regardless of sample matrix, the average THg concentration was less than 1 μg/g dry (mean ± SE; 0.66 ± 0.09 μg/g dry).

**Fig. 1:**
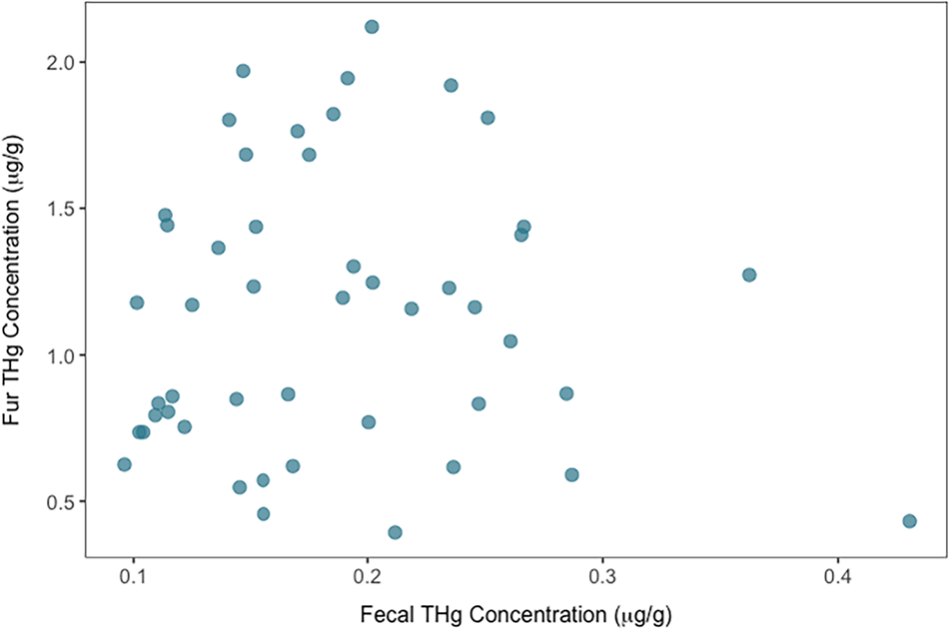
No relationship between individual fecal and fur THg concentrations (dry weight) in *T. brasiliensis*

**Fig. 2:**
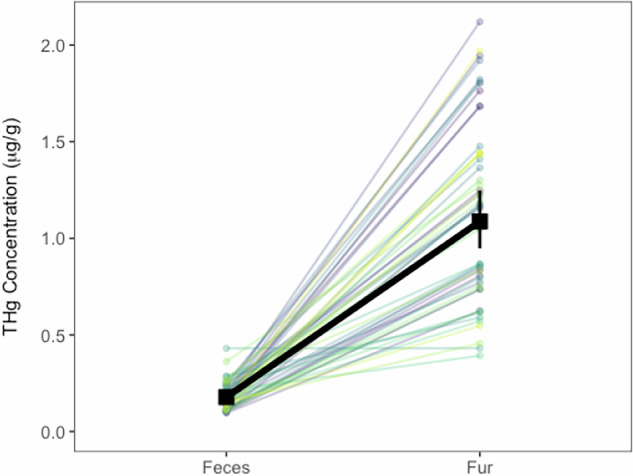
THg concentrations (dry weight) in *T. brasiliensis* fur were six-times greater than those in feces. Fecal and fur samples were paired with each individual, as represented by lighter colored points and lines. Estimated marginal means and 95% confidence intervals of THg concentrations are represented in thicker black points and corresponding lines

Fecal THg ranged from 0.10–0.43 μg/g dry, while fur THg ranged from 0.39–2.12 μg/g dry. We did not find a relationship between individual fecal and fur THg concentrations (χ^2^_1,3_ = 0.26, *P* = 0.61, R^2^_m_ = 0.006, R^2^_c_ = 0.14; Fig. 1). However, when comparing mean THg concentrations across sample matrices at the population level, our GLMM indicated significant differences (χ^2^_1, 47_ = 764.12, *P* < 0.001, R^2^_m_ = 0.83, R^2^_c_ = 0.88; Fig. 2), with concentrations in bat fur being 6.06-times greater than those in feces (marginal mean [lower CI, upper CI]; feces: 0.18 [0.16, 0.20] μg/g dry, fur: 1.09 [0.95, 1.25] μg/g dry, feces/fur contrast: 0.16 ± 0.01 [0.14, 0.18] μg/g dry).

## Discussion

Determining relationships between short- and long-term contaminant exposure for initial risk assessment can benefit wildlife research and conservation. Pooled samples collected indirectly and representing short-term exposure, such as feces obtained under roosts, can cause less disturbance to threatened species than samples requiring direct capture, such as fur. Using paired samples from individual *T. brasiliensis* in the Great Plains of the United States, we did not find a relationship between individual fecal and fur THg concentrations. However, considering population-level means, THg concentrations in bat fur were on average six-times greater than THg concentrations in feces, despite high variability between individual fecal and fur THg concentrations. While these results suggest that THg concentrations in feces cannot be used to predict an individual bat’s fur THg concentration, biologists could cautiously estimate average fur THg concentrations from average fecal THg concentrations to guide initial risk assessment (e.g., from under-roost fecal samples to determine if individual-level health assessment is warranted). However, given the high variability of fecal and fur THg across individuals, we would only suggest such population-level estimates be used in cases where under-roost sampling of feces is preferable to direct capture for species of concern and when funding for individual sampling is initially unavailable.

We did not detect an individual-level relationship in THg concentrations between sample matrices. We attribute this to the greater variability observed in fur THg. First, THg concentrations in fur represent longer-term accumulation timelines than feces (Chételat et al. 2020). The build-up of fur THg concentrations over time will depend on an individual’s foraging area preference, which can be highly variable within the known foraging distance of 40 km for *T. brasiliensis* (Davis et al. 1962), as opposed to the few foraging nights represented by fecal THg. Second, our sample included adult (*n* = 41), subadult (*n* = 1), and juvenile bats (*n* = 5). While sample sizes for subadults and juveniles did not allow for statistical comparisons, different age groups could introduce greater variation in fur THg (Yates et al. 2014). Third, juvenile fur growth occurs in late summer, while there may be more variability in adult and subadult molt and fur growth timing (Fraser et al. 2013). We sampled *T. brasiliensis* during two months in which both adults and juveniles grow fur and thus should represent bioaccumulation. We also accounted for seasonal effects by including sampling date as a random effect. Increasing paired sampling could detect the expected positive correlations between fecal and fur THg and better identify differential relationships between age classes and molt timing across the summer maternity season.

Fecal and fur THg concentrations measured here are similar to concentrations found in other *T. brasiliensis* populations as well as other insectivorous bat species in North America. When averaged, our THg concentrations from *T. brasiliensis* feces and fur were 0.18 μg/g dry and 1.09 μg/g dry, respectively. In the northeastern USA, nine species of insectivorous bats had THg concentrations in fur below 1 μg/g (Yates et al. 2014). *T. brasiliensis* across multiple sites in Texas averaged fur THg concentrations below 2 μg/g (Korstian et al. 2018; Parker et al. 2024). Pooled feces collected in Florida under bat boxes mostly occupied by *T. brasiliensis*, and collected in caves dominated by southeastern myotis (*Myotis austroriparius*), also had average THg concentrations below 1 μg/g (Edwards et al. 2019). Additionally, feces collected below big brown bat (*Eptesicus fuscus*) roosts in Colorado had THg concentrations below 0.3 μg/g (O’Shea et al. 2001). Fur THg values here are also comparable to fur THg concentrations in *E. fuscus* captured at reference sites in the eastern USA (Wada et al. 2010). Similar to these reference values, we are not aware of any immediate point source of Hg in the study region, such that THg concentrations in Great Plains *T. brasiliensis* are likely reflective of atmospheric deposition (Korstian et al. 2018; Chételat et al. 2018). While our THg concentrations in *T. brasiliensis* feces and fur are relatively low, there could still be concern for adverse health effects associated with Hg exposure. Wildlife managers could make decisions regarding a potential THg thresholds for initial risk assessment with under-roost fecal sampling to determine when more invasive fur and health sampling outweighs disturbance risk, particularly for species of conservation concern.

The magnitude of difference between mean fur and fecal THg presented here (an approximately six-fold increase within a confidence margin of 5.94–6.25) could be used as a conversion factor to estimate average fur THg from average fecal THg collected under bat roosts for initial risk assessment, but calculated estimates should be cautiously interpreted. We recognize that the high dispersion of individual-level data from population-level differences is important for making informed decisions regarding follow-up monitoring strategies. Thus, converted estimates for under-roost fecal THg could still bring uncertainty.

In mammals, lethal effects of Hg toxicity typically occur above concentrations of 10 μg/g in tissues such as brain, liver, kidney, and fur (Wolfe et al. 1998; Shore et al. 2011; Nam et al. 2012; Dietz et al. 2021). Thus, a threshold of estimated fur THg around 10 μg/g following under-roost fecal assessment and conversion of mean concentrations by a population-level factor of six (within the 95% confidence interval of 5.94–6.25) could indicate a need for direct health assessment and/or conservation action. However, rough estimates from population-level results should be used cautiously, as the magnitude of difference between average fecal and individual minimum and maximum fur values ranged between 2.17- and 11.78-times. Non-lethal (but still adverse) thresholds of Hg exposure below 10 μg/g are also reported in mammals and bats specifically. For example, Neotropical bat cellular immunity and bacterial killing ability were negatively correlated with fur THg concentrations below 10 μg/g (Becker et al. 2017, 2021). Additionally, in some Neotropical bats, the probability of infection with blood bacteria was associated with higher fur THg, even when concentrations were as low as 0.3 μg/g (Becker et al. 2021). Neotropical bats with spleen and liver THg concentrations between 0.23–0.25 μg/g also had elevated micronuclei intensities in red blood cells (Calao-Ramos et al. 2021), representing genotoxic effects of contaminants (Samanta and Dey 2012; Sandoval-Herrera et al. 2021). All our THg concentrations were below 2.2 μg/g dry (maximum fecal THg: 0.43 μg/g dry; maximum fur THg: 2.12 μg/g dry). To help determine non-lethal thresholds of Hg exposure that may have adverse health effects, future research priorities should include both captive toxicity trials and field experiments assessing immunity, infection, and genotoxic correlates of Hg exposure in this and other insectivorous bat species important to conservation.

We collected paired samples from individuals at capture and did not perform under-roost sampling for bat feces. However, we still suggest under-roost sampling is an applicable method for initial risk assessment from these data. Our mean fecal THg concentrations were similar to concentrations collected under roosts for *T. brasiliensis* and other North American bat species in different locations (O’Shea et al. 2001; Edwards et al. 2019). To ensure future risk assessment methods would be similar to individual sample collection presented here, we recommend managers use stratified sampling methods suggested for under-roost sampling for pathogen surveillance (Giles et al. 2021). We recommend placing multiple cloth sheets or tarps randomly under roosts or at cave entrances to collect feces when bats return from foraging, maximizing the number of sheets versus sheet size depending on the environmental conditions and sampling area. Using cloth sheets limit Hg contamination from soils since feces would not be collected directly from the ground. Combining the collection of pellets for each sheet could then be considered an individually pooled fecal sample for the roost, which would be stored in 95% ethanol and samples completely dried prior to THg analyses (as we did here). If managers cannot deploy sheets/tarps, we recommend sampling the top layer of fecal substrates under the roost, as outlined in O’Shea et al. (2001) to minimize soil Hg contamination. Samples could then be analyzed for THg, and values could be averaged to guide further decisions about whether individual bat sampling is warranted for assessing bat health relative to the risks from roost disturbance.

In this study, fecal and fur THg are representative of *T. brasiliensis* in the Great Plains, USA, with no known Hg point source near our sampling site. THg values in this study are also relatively low, and further research at this site could determine sublethal correlates to bat health in relation to individual THg concentrations. Thus, our assessment may be conditional to similar sites, species, and landscapes with lower THg exposure than bats foraging at sites with higher Hg exposure and risks. For example, insectivorous bats in Malaysia captured near a hydroelectric plant had fur THg concentrations up to 9.5 μg/g (Syaripuddin et al. 2014). Additionally, at a gold mining site in Peru, the highest individual bat fur THg concentration was 8.67 μg/g (Moreno-Brush et al. 2018). *E. fuscus* foraging near a river in Virginia, USA, had a mean fur THg concentration of 28 μg/g at downstream contaminated sites (Wada et al. 2010). In all these examples, individual- and population-level differences between fecal and fur THg could be much greater than our reported values due to known point sources.

## Conclusion

Understanding how contaminant concentrations relate across sampling matrices can inform diverse applications in wildlife conservation research and management. Our study examined both individual- and population-level relationships between fecal and fur THg concentrations in a common insectivorous bat species. Population-level estimates may enable researchers to extrapolate fur concentrations only when fecal samples are the most logistically feasible option for initial risk assessments. Researchers and wildlife managers could use this conversion factor across other insectivorous bat species and sites when species of conservation concern require initial screening-level risk assessment sampling with minimal disturbance; however, we would recommend careful interpretation of calculated fur estimates due to the dispersion of individual fur data. Future research should continue to investigate THg relationships across tissue types, and include quantifying immune and infection correlates to identify the health implications of contaminant exposure and evaluate needs for conservation action.

## Data Availability

All data and R code used for analyses are available on MCS’ Github page (username: simonimc; https://github.com/simonimc/Tadarida_brasiliensis_paired_fecal_fur_THg).
